# FAM19A5, a brain-specific chemokine, inhibits RANKL-induced osteoclast formation through formyl peptide receptor 2

**DOI:** 10.1038/s41598-017-15586-0

**Published:** 2017-11-14

**Authors:** Min Young Park, Hyung Sik Kim, Mingyu Lee, Byunghyun Park, Ha Young Lee, Eun Bee Cho, Jae Young Seong, Yoe-Sik Bae

**Affiliations:** 1 Department of Biological Sciences, Sungkyunkwan University, Suwon, Republic of Korea; 2 Department of Health Sciences and Technology, SAIHST, Sungkyunkwan University, Seoul, Republic of Korea; 3 Graduate School of Medicine, Korea University, Seoul, Republic of Korea

## Abstract

Osteoclasts can be differentiated from bone marrow-derived macrophages (BMDM). They play a key role in bone resorption. Identifying novel molecules that can regulate osteoclastogenesis has been an important issue. In this study, we found that FAM19A5, a neurokine or brain-specific chemokine, strongly stimulated mouse BMDM, resulting in chemotactic migration and inhibition of RANKL-induced osteoclastogenesis. Expression levels of osteoclast-related genes such as *RANK*, *TRAF6*, *OSCAR*, *TRAP*, *Blimp1*, *c-fos*, and *NFATc1* were markedly decreased by FAM19A5. However, negative regulators of osteoclastogenesis such as *MafB* and *IRF-8* were upregulated by FAM19A5. FAM19A5 also downregulated expression levels of RANKL-induced fusogenic genes such as *OC-STAMP*, *DC-STAMP*, and *Atp6v0d2*. FAM19A5-induced inhibitory effect on osteoclastogenesis was significantly reversed by a formyl peptide receptor (FPR) 2 antagonist WRW4 or by FPR2-deficiency, suggesting a crucial role of FPR2 in the regulation of osteoclastogenesis. Collectively, our results suggest that FAM19A5 and its target receptor FPR2 can act as novel endogenous ligand/receptor to negatively regulate osteoclastogenesis. They might be regarded as potential targets to control osteoclast formation and bone disorders.

## Introduction

Family with sequence similarity 19 (chemokine (C-C motif)-like) member A (FAM19A) encoded by *TAFA* genes (*TAFA-1* to *TAFA-5*) are secretory proteins with cytokine-like properties^1,2^. Among these *TAFA* genes, *TAFA* mRNA expression levels are very high in the brain with very low levels in other organs^1^. *TAFA4* has been regarded as a specific marker of C-low-threshold mechanoreceptors^2^. A previous study on the functional role of *TAFA4* has demonstrated that genetic depletion of *TAFA4* can cause severe mechanical and chemical hypersensitivity in response to injury^2^. Another previous report has shown that FAM19A4 encoded by *TAFA4* can promote cellular migration and phagocytosis in macrophages^3^. In addition, FAM19A4 can directly bind to formyl peptide receptor (FPR) 1, its target receptor^3^. FPR1 is a well-known classical chemoattractant receptor for innate immune cells such as monocytes/macrophages and neutrophils^3^. However, the functional role or molecular target receptor of other FAM19A members, especially FAM19A5, has not been reported yet.

Osteoclasts are giant multinucleated cells with bone resorbing activity. They play essential roles in bone metabolism and homeostasis^4,5^. They can adhere to bone surface by interacting with extracellular matrix and degrade bone matrix^6^. Osteoclasts can be differentiated from monocyte/macrophage lineage^7^. Stimulation of macrophages with receptor activation of nuclear factor κB ligand (RANKL) in the presence of macrophage colony-stimulating factor (M-CSF) can elicit osteoclast differentiation^8^. During differentiation of osteoclasts, several osteoclast-associated genes including *RANK*, *TRAF6*, *OSCAR*, *TRAP*, and *NFATc1* are upregulated^4,5^. Because osteoclasts have bone resorbing activity, several bone disorders including osteoporosis and rheumatoid arthritis are associated with enhanced osteoclast formation^9^. Considering the crucial role of osteoclasts in bone diseases, identifying molecules that can inhibit osteoclast differentiation is essential to control these diseases. In this study, we found that FAM19A5 stimulated mouse bone-marrow-derived macrophages (BMDMs) which could be differentiated into osteoclasts, leading to chemotactic migration of cells. We further investigated whether FAM19A5 could affect osteoclast formation from mouse BMDMs. Interestingly, we found that FAM19A5 strongly inhibited RANKL-induced osteoclastogenesis. Target receptor and signaling pathways involved in these processes are also examined in this study.

## Results

### FAM19A5 stimulates BMDM, leading to chemotactic migration via FPR2

**Figure 1:**
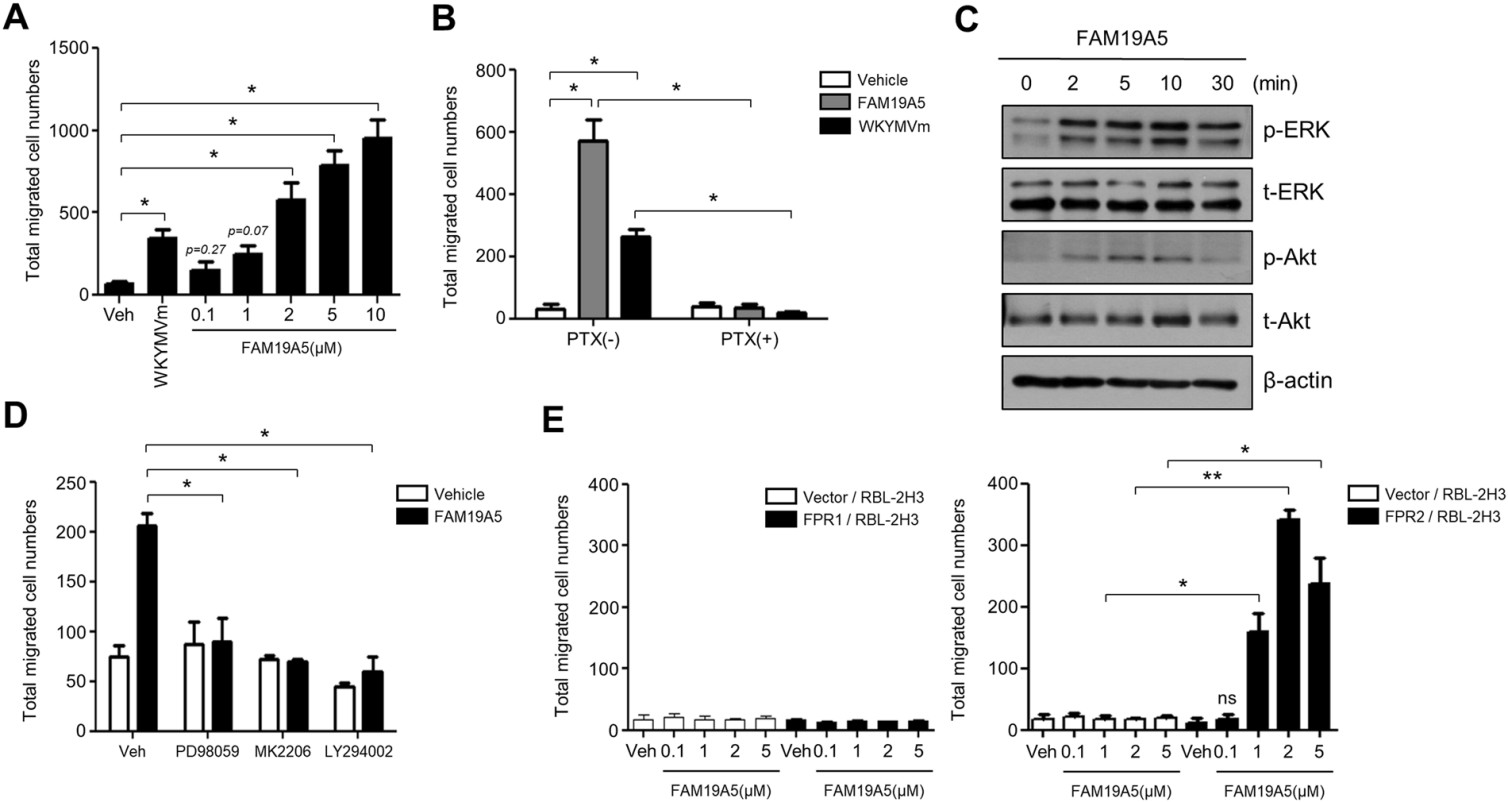
FAM19A5 stimulates BMDM chemotaxis via FPR2. (**A**) Mouse BMDMs were used for chemotaxis assay using multiwell chamber containing several concentrations (0, 0.1, 1, 2, 5, 10 μM) of FAM19A5 or 1 μM of WKYMVm for 2 h. (**B**) Mouse BMDMs were incubated in the absence or presence of 500 ng/ml PTX for 4 h and applied to the upper well of the multiwell chamber containing 2 μM of FAM19A5 or 1 μM of WKYMVm for 2 h. (**C**) Mouse BMDMs were stimulated with 2 μM of FAM19A5 for 0, 2, 5, 10, and 30 min. Total cell lysates were separated by SDS-PAGE. Levels of p-ERK and p-Akt were measured by Western blot analysis. Data are representative of three independent experiments (**C**). (**D**) Mouse BMDMs were incubated in the absence or presence of PD98059 (50 μM) for 60 min, LY294002 (50 μM) for 15 min, or MK-2206 (2 μM) for 20 min and applied to the upper well of the multiwell chamber containing 2 μM of FAM19A5 for 2 h. (**E**) Vector-, FPR1-, or FPR2- expressing RBL-2H3 cells were applied to the upper well of a multiwell chamber containing 2 μM of FAM19A5 for 4 h. The number of migrated cells was determined by counting under a light microscope (**A**,**B**,**D**,**E**). Data are presented as means ± SE (n = 3). Data are representative of at least three independent experiments. ns: not significant, *p < 0.05; **p < 0.01.

It has been reported that FAM19A4 possesses cytokine-like property and stimulates macrophage chemotaxis^3^. In this study, we tested whether FAM19A5 could stimulate macrophage activity, especially chemotactic migration using Boyden chamber assay. FAM19A5 strongly stimulated chemotactic migration of BMDM, showing maximal activity at 10 μM (Fig. 1A). These results suggest that BMDMs are activated by FAM19A5. Chemokines and chemoattractant are known to stimulate macrophage chemotaxis through pertussis toxin (PTX)-sensitive G-protein(s)^10^. Our results showed that FAM19A5-induced BMDM chemotaxis was significantly blocked by PTX (Fig. 1B). As a control experiment, we found that WKYMVm (an agonist for FPR members)-stimulated BMDM chemotaxis was completely inhibited by PTX (Fig. 1B). These results suggest that FAM19A5 can stimulate BMDM chemotaxis via PTX-sensitive G-protein(s). Activation of BMDM by diverse extracellular stimuli can induce the activation of intracellular signaling kinases such as ERK and Akt^11,12^. Stimulation of BMDM with FAM19A5 also induced phosphorylation of ERK and Akt in a time-dependent manner, suggesting that FAM19A5 could stimulate ERK and Akt activities (Fig. 1C). FAM19A5-stimulated ERK phosphorylation was apparent at 2-30 min after stimulation. However, Akt phosphorylation was induced at 2–10 min. It then returned to its basal level after the stimulation (Fig. 1C). We then examined whether these ERK and Akt activities were required for FAM19A5-stimulated BMDM chemotaxis using specific inhibitors of kinases. FAM19A5-induced BMDM chemotaxis was almost completely inhibited by PD98059 (an ERK inhibitor), MK2206 (an Akt inhibitor), and LY294002 (a PI3K inhibitor) (Fig. 1D). These results suggest that FAM19A5-induced BMDM chemotaxis is mediated by ERK and Akt pathway.

In a previous report, FAM19A4 has been demonstrated to be able to bind FPR1 and promote macrophage chemotaxis and phagocytosis^3^. In this study, we examined whether FAM19A5 could act on FPR family using vector-, FPR1-, or FPR2- expressing RBL-2H3 cells generated previously^13^. Stimulation of FPR2-expressing RBL-2H3 cells with FAM19A5 elicited chemotactic migration of these cells (Fig. 1E right). However, FAM19A5 failed to induce cellular migration of vector- or FPR1- expressing RBL-2H3 cells (Fig. 1E left). These results suggest that FAM19A5 can stimulate macrophage chemotaxis through FPR2, but not through FPR1. Collectively, we found that FAM19A5, a brain-specific chemokine, was a novel endogenous regulator of macrophage activity.

### FAM19A5 inhibits osteoclast formation from mouse BMDMs

**Figure 2:**
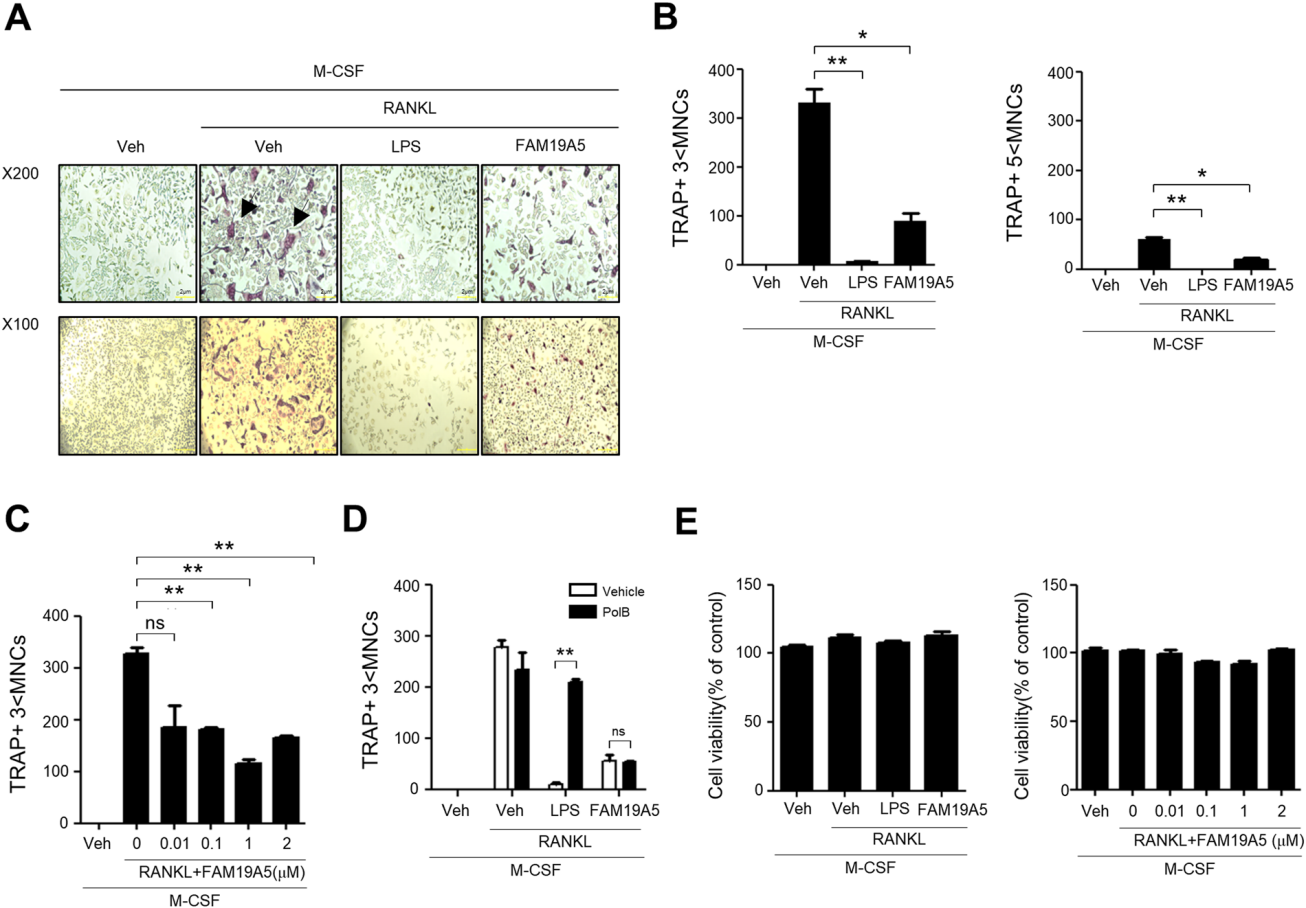
FAM19A5 blocks RANKL-induced osteoclastogenesis. (**A**,**B**) Mouse BMDMs were stimulated with FAM19A5 (2 μM) or LPS in the presence of M-CSF (30 ng/ml) and RANKL (100 ng/ml) for 4 days. All cells were stained with TRAP staining solution. TRAP+ 3 < MNCs (B, left) and TRAP+ 5 < MNCs (B, right) were counted. (**C**) Mouse BMDMs were stimulated with various concentrations of FAM19A5 (0, 0.01, 0.1, 1, and 2 μM) in the presence of M-CSF (30 ng/ml) and RANKL (100 ng/ml) for 4 days. (**D**) Mouse BMDMs were pre-incubated with polymyxin B (10 μM) prior to FAM19A5 (2 μM) and LPS (1 μg/ml) treatment in the presence of M-CSF (30 ng/ml) and RANKL (100 ng/ml) for 4 days. (**E**) Mouse BMDMs were stimulated with 2 μM FAM19A5 (left) or various concentrations of FAM19A5 (0, 0.01, 0.1, 1, and 2 μM) (right) in the presence of M-CSF (30 ng/ml) and RANKL (100 ng/ml) for 4 days. After adding CCK-8 assay solution, the OD was measured at 450 nm. Data in these panels are representative of three independent experiments (**A**). Data are presented as means ± SE (n = 3). Data are representative of at least three independent experiments (**B**–**E**). ns: not significant, *p < 0.05; **p < 0.01.

Our finding on the stimulatory effect of FAM19A5 on BMDM chemotactic migration led us to examine additional effects of FAM19A5 on BMDMs. BMDMs can be differentiated into osteoclasts which play an essential role in bone metabolism^4,5,7^. In this study, we examined whether FAM19A5 affected osteoclastogenesis using mouse BMDMs. Isolated mouse bone marrow cells were differentiated into BMDM by adding M-CSF. Incubation of BMDMs with M-CSF and RANKL-induced osteoclast formation based on tartrate-resistant acid phosphatase (TRAP) staining (Fig. 2A). Interestingly, addition of FAM19A5 strongly blocked osteoclast formation from BMDMs (Fig. 2A). Quantitation of osteoclast formation showed that FAM19A5 inhibited RANKL–induced osteoclast (TRAP + 3 < MNCs) formation by 73.23% (Fig. 2B left). Lipopolysaccharide (LPS), a well-known osteoclast formation inhibitory molecule, almost completely blocked RANKL-induced osteoclast formation (Fig. 2B left). FAM19A5 also inhibited TRAP + 5 < MNCs cell number by approximately 67.22% (Fig. 2B right). Several different concentrations of FAM19A5 were used to test their inhibitory effects on osteoclast formation. Addition of 0.1–2 μM of FAM19A5 markedly blocked osteoclast formation (Fig. 2C). Since FAM19A5 is a recombinant protein expressed in *E*. *coli*, we investigated if the osteoclast formation inhibitory effect of FAM19A5 could be induced by LPS endotoxin using polymyxin B, an endotoxin inhibitor. FAM19A5-induced inhibitory effects on osteoclast formation was not affected by polymyxin B (Fig. 2D). However, LPS-induced osteoclast formation inhibitory effect was blocked by polymyxin B (Fig. 2D). We also investigated whether the inhibitory effect of FAM19A5 on osteoclast formation was mediated by cell death during osteoclastogenesis. Addition of FAM19A5 failed to affect cell viability under osteoclastogenesis condition (Fig. 2E). These results suggest that FAM19A5 can block osteoclast formation from BMDMs without affecting cell viability.

### FAM19A5 regulates the expression of RANKL-induced osteoclast-related genes

**Figure 3:**
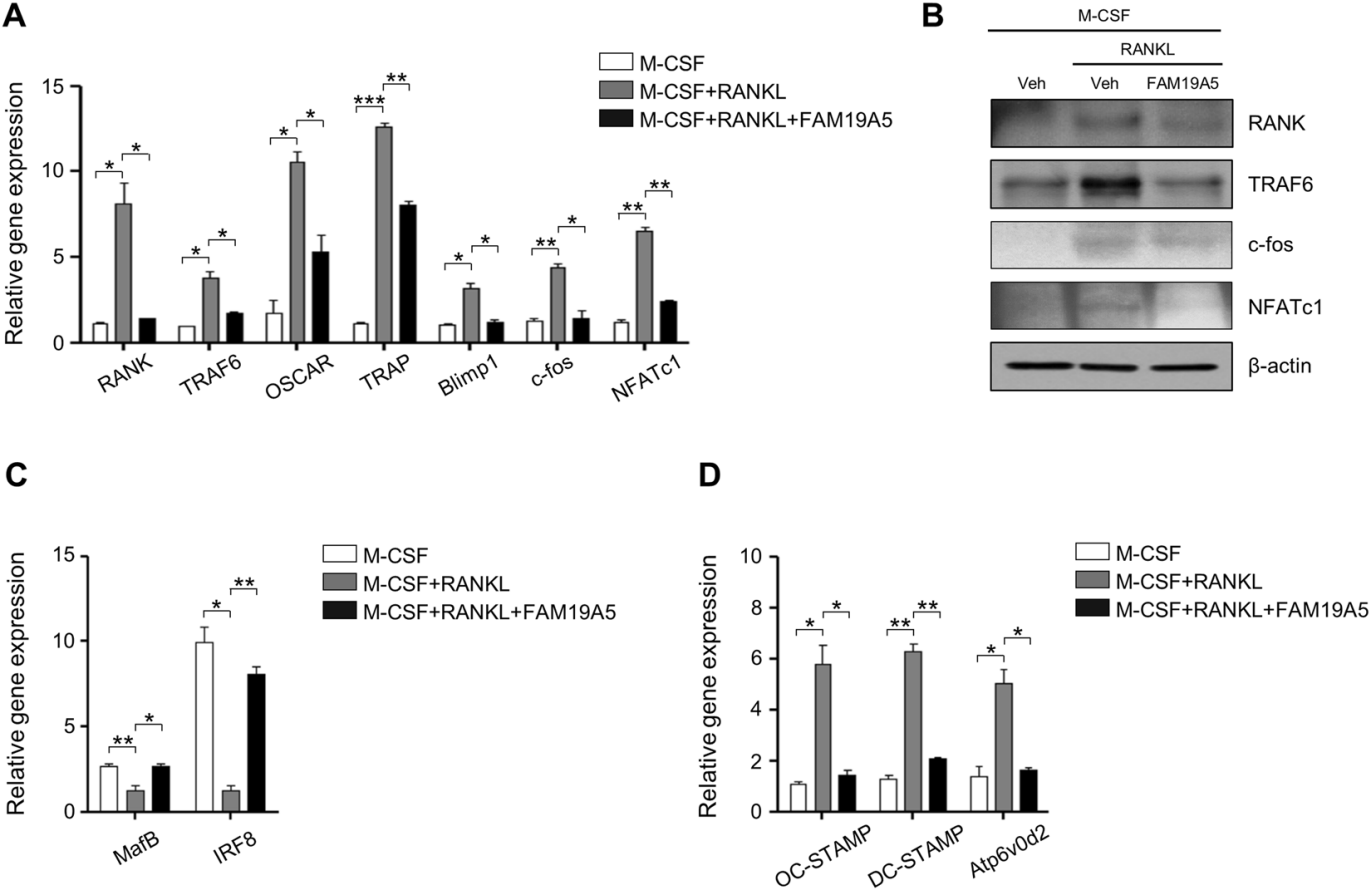
FAM19A5 regulates RANKL-induced gene expression during osteoclastogenesis. (**A**,**C**,**D**) Mouse BMDMs were stimulated with FAM19A5 (2 μM) in the presence of M-CSF (30 ng/ml) and RANKL (100 ng/ml) for 3 days. Cells were harvested for RNA preparation. qPCR was performed using specific primers for *RANK*, *TRAF6*, *OSCAR*, *TRAP*, *Blimp1*, *c-FOS*, *NFATc1*, *MafB*, *IRF-8*, *DC-STAMP*, *OC-STAMP*, *Atp6v0d2*, and *GAPDH*. (**B**) Cells were harvested and Western blot analysis was conducted using anti-RANK, anti-TRAF6, anti-c-fos, anti-NFATc1, and β-actin antibodies. Data are presented as means ± SE (n = 3). Data are representative of three independent experiments (**A**,**C**,**D**). *p < 0.05; **p < 0.01; ***p < 0.001. Results shown are representative of three independent experiments (**B**).

Since osteoclasts are specialized cells differentiated from BMDMs, differentiated osteoclast are known to express several osteoclast-related genes^14,15^. Previous studies have reported that osteoclasts express *RANK*, *TRAF6*, *OSCAR*, *TRAP*, and *NFATc1* ^16,17^. In this study, we also found that differentiation of osteoclast from BMDMs by RANKL upregulated the expression of these osteoclast-related genes by qPCR analysis (Fig. 3A). RANKL-induced upregulation of these osteoclast-related genes was markedly inhibited by FAM19A5 (Fig. 3A). Protein expression of RANK, TRAF6, c-fos, and NFATc1 was also induced by RANKL. However, such effect of RANKL was markedly blocked by FAM19A5 (Fig. 3B). MafB and IRF-8 are well-known negative regulators of osteoclast formation^18,19^. Stimulation of BMDMs with RANKL elicited downregulated expression of *MafB* and *IRF8* (Fig. 3C). However, addition of FAM19A5 blocked the downregulation of these two negative regulators of osteoclast formation (Fig. 3C).

During osteoclast differentiation, osteoclast precursors need to be fused together to generate immature and mature osteoclasts^20^. The fusion process is regulated by several genes, including *dendritic cell-specific transmembrane protein* (*DC-STAMP*), *osteoclast stimulatory transmembrane protein* (*OC-STAMP*), and *Atp6v0d2* ^21,22^. In this study, we also observed that expression levels of *DC-STAMP*, *OC-STAMP*, and *Atp6v0d2* were markedly increased in the presence of M-CSF plus RANKL in BMDMs by qPCR analysis (Fig. 3D). We then tested the effect of FAM19A5 on the expression of these fusogenic genes. The addition of FAM19A5 significantly decreased expression levels of these osteoclast fusogenic genes (Fig. 3D). Collectively, these results suggest that FAM19A5 can regulate the expression of osteoclast differentiation and fusion-associated genes, leading to inhibition of osteoclastogenesis.

### FPR2 mediates FAM19A5-induced inhibitory effects on osteoclast formation

**Figure 4:**
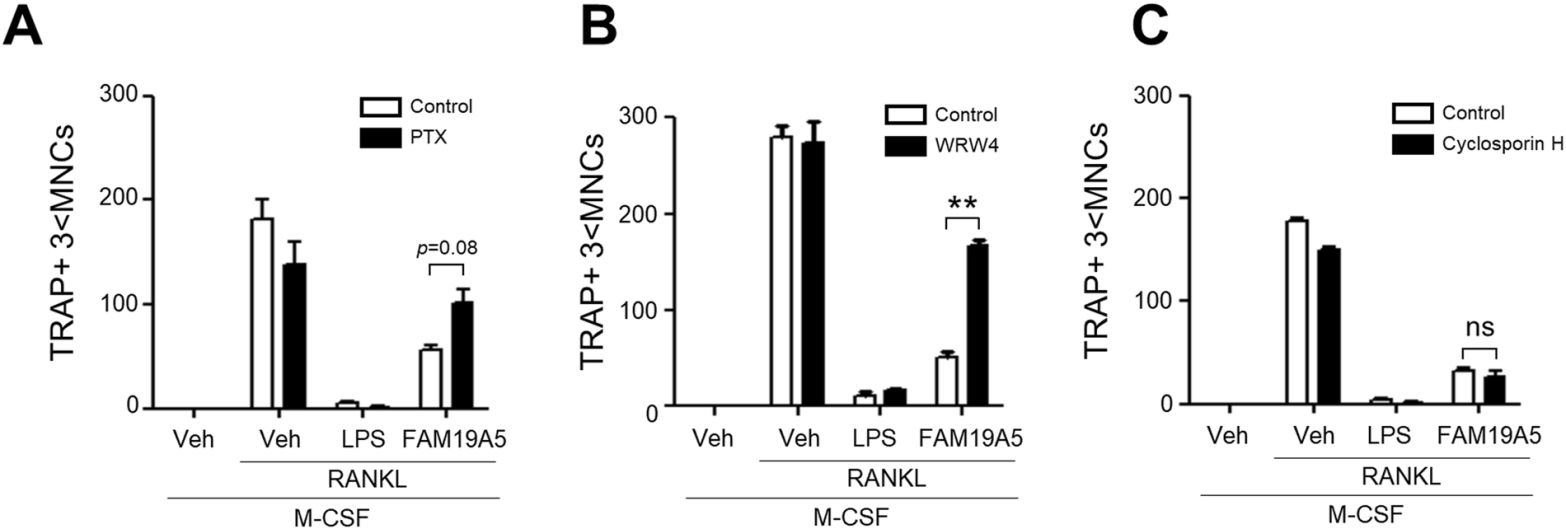
Inhibitory effects of FAM19A5 on RANKL-induced osteoclast formation are mediated by FPR2. (**A**–**C**) Mouse BMDMs were pre-incubated with PTX (100 ng/ml), WRW4 (60 μM) or cyclosporin H (10 μM) prior to FAM19A5 (2 μM) treatment in the presence of M-CSF (30 ng/ml) and RANKL (100 ng/ml) for 4 days. All cells were stained with TRAP staining solution. Osteoclasts were considered for TRAP+ 3 < MNCs. Data are presented as means ± SE (n = 3). Data are representative of at least two independent experiments. ns: not significant, **p < 0.01.

Since we found that FAM19A5 could stimulate BMDM chemotaxis via FPR2, we investigated whether FPR2 played a role in FAM19A5-induced inhibition of osteoclast formation. Cellular signaling of FPR2 is mediated by PTX-sensitive G-proteins^23^. Therefore, we tested the effect of PTX on FAM19A5-induced inhibitory effect on osteoclast formation. Pre-incubation of BMDMs with PTX (which inactivates G proteins) prior to the addition of FAM19A5 reversed FAM19A5-induced inhibitory effect on osteoclast formation (Fig. 4A). These results suggest that FAM19A5-induced osteoclastogenesis inhibitory effect is mediated by PTX-sensitive G proteins. We then investigated the role of FPR2 on FAM19A5′s inhibitory effect on osteoclast formation using WRWWWW (WRW4)^24^, an FPR2 antagonist. FAM19A5-induced inhibitory effect on osteoclast formation was significantly reversed by WRW4 (Fig. 4B). However, LPS-induced inhibitory effect on osteoclast formation was not affected by WRW4 (Fig. 4B). In addition to FPR2, FPR1 is another member of FPR that plays a crucial role in the regulation of leukocyte chemotactic migration^25^. We also tested the role of FPR1 on FAM19A5-induced inhibitory effect on osteoclast formation. Pre-incubation of BMDMs with cyclosporin H, an FPR1 antagonist, prior to addition of FAM19A5 failed to affect FAM19A5-induced inhibitory effect on osteoclast formation (Fig. 4C). These results suggest that FAM19A5-induced osteoclast formation inhibition is mediated by FPR2, but not by FPR1.

### FPR2-deficiency blocks FAM19A5-induced macrophage activation but reverses inhibitory effect of FAM19A5 on osteoclast formation

**Figure 5:**
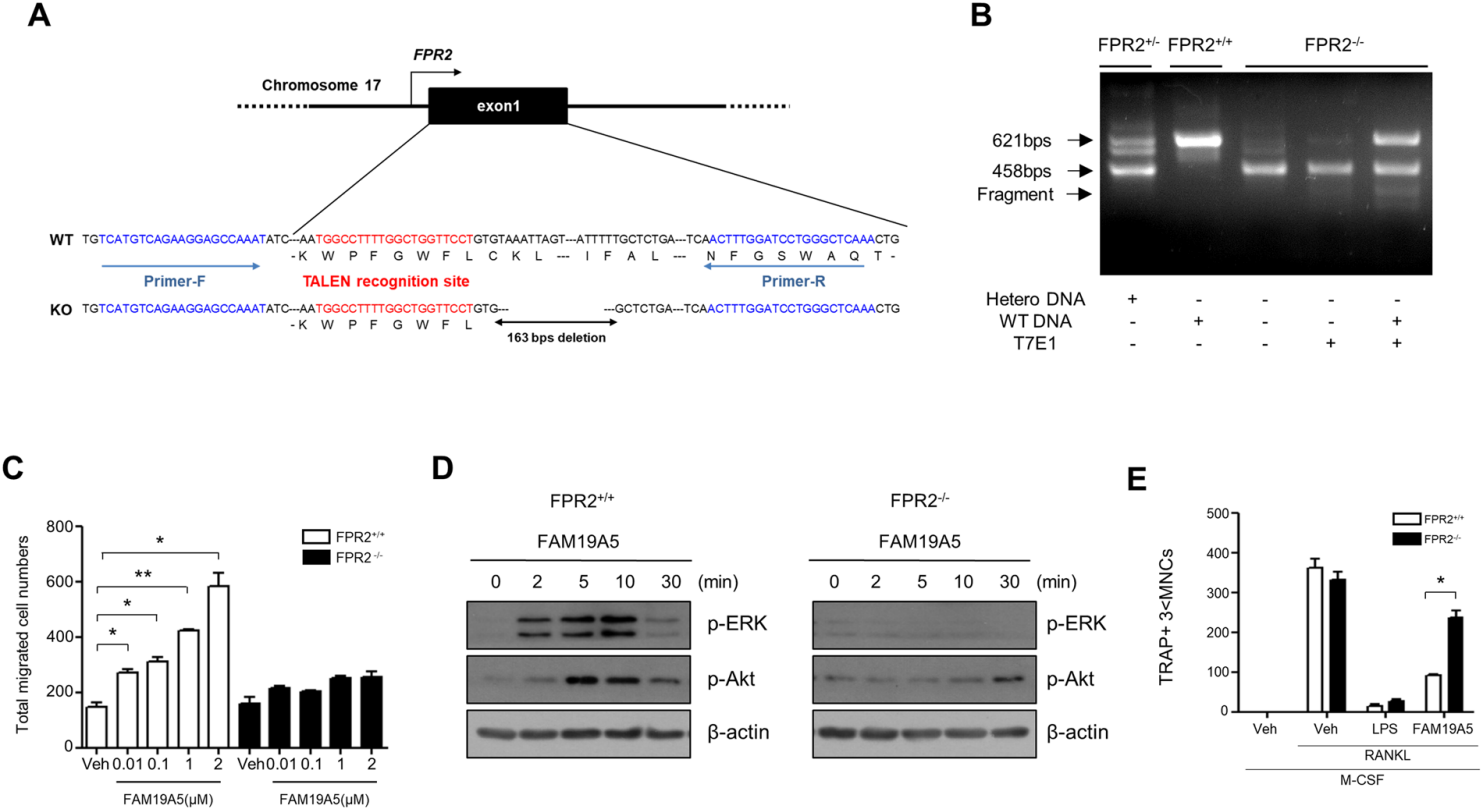
FPR2-deficiency blocks FAM19A5-induced BMDM chemotaxis and FAM19A5′s inhibitory effects on osteoclastogenesis. (**A**) TALEN recognition site for the generation of FPR2 knockout mice. (**B**) T7E1 assay of WT, heterozygotes, and homozygotes of FPR2 knockout mice. (**C**) BMDMs generated from FPR2^+/+^ or FPR2^−/−^ mice were subjected to chemotaxis assay using several concentrations (0, 0.01, 0.1, 1, and 2 μM) of FAM19A5. The number of migrated cells was determined by counting under a light microscope. (**D**) Mouse BMDMs generated from FPR2^+/+^ or FPR2^−/−^ mice were stimulated with 2 μM of FAM19A5 for 0, 2, 5, 10, and 30 min. Total cell lysates were separated by SDS-PAGE. Levels of p-ERK and p-Akt were measured by Western blot analysis. (**E**) Mouse BMDMs were stimulated with FAM19A5 (2 μM) in the presence of M-CSF (30 ng/ml) and RANKL (100 ng/ml) for 4 days. All cells were stained with TRAP staining solution. The number of cells with TRAP+ 3 < MNCs was counted. Data in these panels are representative of three independent experiments (**D**). Data are presented as means ± SE (n = 3). Data are representative of three independent experiments (**C**,**E**). *p < 0.05; **p < 0.01.

To support our notion that FPR2 could mediate FAM19A5-induced macrophage activation and osteoclast differentiation inhibition, we generated FPR2 knockout (FPR2^−/−^) mice by TALEN-mediated gene targeting (Fig. 5A). *FPR2* gene knockout was confirmed by T7E1 assay (Fig. 5B). Since we found that FAM19A5 stimulated BMDM chemotaxis through ERK and Akt pathway, we investigated whether FPR2-deficiency affected FAM19A5-induced BMDM chemotaxis and ERK/Akt phosphorylation using BMDM from FPR2^+/+^ or FPR2^−/−^ mice. As expected, we observed that FAM19A5-stimulated BMDM chemotaxis and ERK/Akt phosphorylation were strongly blocked by FPR2 deficiency (Fig. 5C,D). We also tested the effect of FPR2 deficiency on FAM19A5-induced inhibitory effect on osteoclast formation. FAM19A5′s inhibitory effect on RANKL-induced osteoclast differentiation was significantly reversed by FPR2 deficiency (Fig. 5E). Taken together, these results strongly indicate that FPR2 plays an essential role in FAM19A5-induced macrophage chemotaxis, ERK/Akt phosphorylation, and FAM19A5′s inhibition on osteoclastogenesis.

## Discussion

Although several previous reports have shown that FAM family members are involved in some biological functions, including the regulation of leukocyte adhesion, migration^3,26^, phagocytosis^3^, vascular inflammation^27^, and microglia/macrophage polarization^28^, studies on the functional role of FAM19A5 are limited. In this study, we demonstrated that FAM19A5, an endogenous brain-specific chemokine, strongly inhibited RANKL-induced osteoclast generation, suggesting a novel inhibitory functional role of FAM19A5 in osteoclastogenesis. Since osteoclasts play essential roles in bone metabolism by mediating bone resorption, resulting in bone disorders such as osteoporosis and rheumatoid arthritis, our findings provide a novel insight on the pathophysiological role of FAM19A5 in bone metabolism and bone-related disorders.

Regarding FAM19A5′s inhibitory effects against osteoclast formation, we demonstrated that FPR2, its target receptor, mediated the process. FAM19A5-inhibited osteoclastogenesis was blocked by WRW4, an FPR2 antagonist (Fig. 4B). Moreover, the inhibitory effect of FAM19A5 on RANKL-induced osteoclastogenesis was not apparent in BMDMs generated from FPR2-deficient mice (Fig. 5E). These results strongly indicate that FAM19A5 inhibits RANKL-induced osteoclast differentiation by stimulating FPR2. Several previous reports have reported that FPR2 play an essential role in the regulation of macrophage/monocyte differentiation into other cells such as foam cells^29–31^, dendritic cells^32^, and macrophage polarization^33^. We have previously demonstrated that FPR2 can mediate cellular differentiation such as foam cell formation induced by oxLDL or serum amyloid A (SAA)^29,30^. FPR2 can also mediate immune-modulating peptide WKYMVm-induced dendritic cell maturation inhibition^32^. Li *et al*. have demonstrated that FPR2 agonists SAA and LL-37 can elicit M2-like macrophage polarization^33^. This process is mediated by FPR2^33^. However, in this study, we report that FPR2 is involved in the regulation of osteoclastogenesis. The role of FPR2 in FAM19A5-induced cellular response was also observed in this study because FAM19A5 stimulated macrophage chemotactic migration which was blocked by PTX, a G_i_-protein inhibitor. Moreover, we demonstrated that FAM19A5 stimulated chemotactic migration of FPR2-expressing RBL-2H3 cells, but not that of vector-expressing RBL-2H3 cells (Fig. 1), indicating that FPR2 could mediate FAM19A5-induced chemotactic migration. FAM19A5-stimulated BMDM chemotaxis and ERK/Akt phosphorylation were also markedly attenuated by FPR2 deficiency (Fig. 5C,D). These results strongly support the notion that FPR2 mediates FAM19A5-induced biological functions such as inhibition of osteoclastogenesis.

Through studying the mechanism of action involved in the inhibitory effect of FAM19A5 on RANKL-induced osteoclastogenesis, we demonstrated that FAM19A5 could modulate gene expression induced by RANKL. RANKL-induced osteoclast-associated genes (*RANK*, *TRAF6*, *OSCAR*, *TRAP*, *Blimp1*, *c-fos*, and *NFATc1*) were downregulated by FAM19A5. However, negative regulator genes of osteoclastogenesis were upregulated by FAM19A5 (Fig. 3A–C). Osteoclast fusogenic genes were strongly decreased by FAM19A5 (Fig. 3D). Since FAM19A5-induced inhibitory effect on osteoclastogenesis was mediated by FPR2, these results suggested that there might be cross-talks between FAM19A5-stimulated FPR2 signaling and RANKL-induced RANK signaling in macrophages. FAM19A5-induced FPR2 signaling might block RANK signaling, resulting in inhibition of osteoclast-associated genes expression and increased negative regulator of osteoclast genes. In a previous report, we have demonstrated that SAA can block osteoclast formation by stimulating c-fms shedding^34^. Therefore, we also examined the effect of FAM19A5 on c-fms shedding and found that stimulation of BMDMs with FAM19A5 did not induce c-fms shedding (data not shown). These results suggest that FAM19A5 blocks osteoclast differentiation by mechanism different from SAA-induced one, at least partly.

Although FAM19A5 was known to be a brain-specific chemokine, we found that FAM19A5 negatively regulated osteoclast differentiation from macrophages. It has been previously reported that other FAM19 members such as FAM19A3 and FAM19A4 can regulate macrophage activity^3,28^. Considering our findings and others’ findings, FAM19 members might be involved in the regulation of peripheral immune cell activity, especially macrophages. Because studies on expression of FAM19A5 are limited, it is currently unclear whether the expression level of FAM19A5 will change during specific pathological progress. A recent proteomics study has reported that FAM19A5 level is differentially expressed in cholangiocarcinoma compared to that in benign biliary tract diseases^27^. In this study, we could not detect any expression of FAM19A5 in BMDM or osteoclast through RT-PCR analysis (data not shown). However, since BMDM express target receptor for FAM19A5, FPR2, these cells can be activated by FAM19A5 to mediate biological function. Future studies are needed to determine the expression of FAM19A5 in specific disease conditions to further understand the biological role of FAM19A5 in diseases. Our findings provide important insights to the pathophysiological role of FAM19A5 in disease states.

In conclusion, we found that stimulation of BMDMs with FAM19A5, a member of brain-specific chemokine, strongly blocked RANKL-induced osteoclast differentiation. We demonstrated that FAM19A5-induced inhibition of osteoclastogenesis was mediated by FPR2. Taken together, our results provide novel insights on the functional role of FAM19A5 and its target receptor FPR2 as crucial candidate for modulating osteoclast formation and bone disorders.

## Materials and Methods

### Materials

Human recombinant FAM19A5 was purchased from Biovendor (Brno, Czech Republic). Murine recombinant M-CSF and murine RANKL were purchased from Peprotech (Rocky Hill, NJ, USA). LPS was purchased from Sigma-Aldrich (St. Louis, MO, USA). WKYMVm and WRW4 were synthesized from Anygen (Gwangju, Korea). Polymyxin B, PTX, and PD98059 were purchased from Calbiochem (San Diego, CA, USA). LY294002 was obtained from BIOMOL Research Laboratories, Inc. (Plymouth Meeting, PA, USA). MK-2206 was purchased from Selleck Chemicals (Houston, TX, USA). Phospho-Akt (Ser473), Akt, phospho-ERK, ERK, RANK, TRAF6, and β-actin antibodies were purchased from Cell Signaling Technology (Beverly, MA, USA). c-fos and NFATc1 antibodies were purchased from Santa Cruz Biotechnology (Santa Cruz, CA, USA).

### Mouse BMDM generation

All animal experiments were performed in accordance with the Korea Food and Drug Administration guidelines. Protocols were approved by the Animal Care and Use Committee, Sungkyunkwan University. Mouse BMDMs were generated according to a previous report^35^. Briefly, bone marrow cells were isolated by flushing femurs and tibias of wild-type C57BL/6 mice of five to seven weeks of age with ice-cold PBS. Bone marrow progenitor cells were cultured in α-MEM supplemented with 10% fetal bovine serum (FBS) for one day. After harvesting non-adherent cells, they were cultured for three days with α-MEM supplemented with 10% FBS and 30 ng/ml M-CSF.

### Chemotaxis assay

Chemotaxis assays were performed using multiwell chambers (Neuroprobe Inc., Gaithersburg, MD, USA) according to a previous report^36^. Briefly, generated mouse BMDMs were suspended in opti-MEM medium at a concentration of 1 × 10^6^ cells/ml and 25 μl was placed onto the upper well of a chamber separated by a 5-μm polyhydrocarbon filter from a FAM19A5-containing lower well. Polycarbonate filters (8-μm pore size) precoated with 50 μg/ml rat type I collagen (Collaborative Research, Bedford, MA, USA) in DMEM medium were used for chemotaxis assay using vector-, FPR1-, or FPR2- expressing RBL-2H3 cells. After incubation at 37°C for 2 h (4 h for vector-, FPR1-, or FPR2-expressing RBL-2H3 cells), non-migrated cells were removed by scraping. Cells that migrated across the filter were dehydrated, fixed, and stained with hematoxylin (Sigma-Aldrich, St. Louis, MO, USA). Stained cells in each well were counted under a light microscope.

### Osteoclast differentiation and TRAP staining

Mouse BMDMs (1 × 10^4^ cells/well) were differentiated into osteoclasts using M-CSF (30 ng/ml) and RANKL (100 ng/ml) according to a previous report^37^. Differentiated osteoclasts were stained for TRAP using acid phosphatase leukocyte diagnostic kit (Sigma-Aldrich, St. Louis, MO, USA) as described previously^34^. Stained cells in five randomly chosen fields (200 × magnification) were then counted for each well.

### Cell viability assay

Cell viability assay was conducted using cell counting kit-8 solution (Dojindo, Rockville, MD, USA) as described previously^38^.

### Quantitative polymerase chain reaction (qPCR) analysis

qPCR was designed based on consensus sequences of each alignment and performed using Rotor-gene Q (2plex on PC) instrument (QIAGEN; Hilden, Germany) with SYBR Green qPCR Mix (Biofact). The following primers were used for qPCR: *RANK-*forward, 5′-AGAAGACGGTGCTGGAGTCT-3′; *RNAK-*reverse, 5′-TAGGAGCAGTGAACCAGTCG-3′; *TRAF6-*forward, 5′-GCCCAGGCTGTTCATAATGT-3′; *TRAF6*-reverse, 5′-TCGCCCACGTACATACTCTG-3′; *OSCAR-*forward, 5′-CTGCTGGATACGGATCAGCTCCCCAGA-3′; *OSCAR*-reverse, 5′-CCAAGGAGCCAGAACCTTCGAAACT-3′; *TRAP-*forward, 5′-CAGTTGGCAGCAGCCAAGGAGGAC-3′; *TRAP*-reverse, 5′-TCCGRGCTCGGCGATGGACCAGA-3′; *Blimp1-*forward, 5′-TGCTTATCCCAGCACCCC-3′; *Blimp1-*reverse, 5′- CTTCAGGTTGGAGAGCTGACC -3′; *c-fos-*forward, 5′-AGAGCGGGAATGGTGAAGAC-3′; *c-fos-*reverse, 5′-GCTGCATAGAAGGAACCGGA-3′; *NFATc1-*forward, 5′- CAACGCCCTGACCACCGATAG -3′; *NFATc1-*reverse, 5′-GGGAAGTCAGAAGTGGGTGGA-3′; *MafB*-forward, 5′-AGTGTGGAGGACCGCTTCTCT-3′; *MafB-*reverse, 5′-CAGAAAGAACTCAGGAGAGGAGG-3′; *IRF-8-*forward, 5′AGACGAGGTTACGCTGTGC-3′; *IRF-8-*reverse, 5′- TCGGGGACAATTCGGTAAACT -3′. Data were normalized against the expression of *GAPDH* as an internal control.

### Western blot assay

Cell extracts were prepared in lysis buffer containing 20 mM HEPES (pH7.2), 10% glycerol, 150 mM NaCl, 1% Triton X-100, 50 mM NaF, 1 mM Na_3_VO_4_, 10 μg/ml leupeptin, 10 μg/ml aprotinin, and 1 mM PMSF. Western blot analysis was conducted as described previously^39^.

### FPR2 knockout mouse generation, maintenance, and genotyping

FPR2 knockout mice were generated as described previously^40^. Briefly, TALENs specific for FPR2 were microinjected into fertilized embryos with C57BL/6 N background. Mutations on *FPR2* were screened by T7E1 assay. Mutant mouse line with 163 bp deletion was selected and confirmed by Sanger sequencing (Cosmo Genetech). Mice were maintained on a normal diet (PicoLab® Rodent Diet 20, Orientbio) under a 12 h light/dark cycle. Genotypes of mice were determined by PCR with following primers; 5′-TCAGGTGCAGACAAAATGGA-3′ (forward) and 5′-TTTGAGCCCAGGATCCAAAGT-3′ (reverse).

### Statistical analysis

Results were analyzed with GraphPad prism software (GraphPad Software, Inc., La Jolla, CA, USA). Statistical analysis was performed using one-way analysis of variance followed by Student’s *t*-test. All results are expressed as mean ± SE. A *p* value < 0.05 was considered statistically significant.

### Data availability statement

No datasets were generated or analysed during the current study.
